# Supplementary material for: Heat adaptation of phage T7 under an extended genetic code
Source: Virus Evol. 2021 Dec 1;7(2):veab100. doi: 10.1093/ve/veab100 (PMC8923235; doi:10.1093/ve/veab100)
Supplement: veab100_Supp [file veab100_supp.zip › Sequencing_Analysis.zip!/Sequencing_Analysis/S1/evidence/JC_SIDE_2_54.html]

BRESEQ :: Evidence


| Predicted mutation | | | | | | |
| --- | --- | --- | --- | --- | --- | --- |
| evidence | seq id | position | mutation | annotation | gene | description |
| MC JC | NC\_001604.1 | 13,569 | Δ770 bp | intergenic (–/–) | – / – | –/– |

| Missing coverage evidence... | | | | | | | | | | |
| --- | --- | --- | --- | --- | --- | --- | --- | --- | --- | --- |
|  |  |  | seq id | start | end | size | ←reads | reads→ | gene | description |
| \* | \* | ÷ | NC\_001604.1 | 13569 | 14338 | 770 | 4673 [104] | [4] 4561 | *–/–* | –/– |

| New junction evidence | | | | | | | | | | | |
| --- | --- | --- | --- | --- | --- | --- | --- | --- | --- | --- | --- |
|  | | seq id | position | reads (cov) | reads (cov) | score | skew | freq | annotation | gene | product |
| \* | ? | NC\_001604.1 | = 13568 | 104 (0.020) | 4361 (1.060) | 369/586 | 0.2 | 98.8% | intergenic (–/–) | *–/–* | –/– |
|  | ? | NC\_001604.1 | 14339 = | 1 (0.000) |  |  |  |  | intergenic (–/–) | *–/–* | –/– |

Only 100 of 4561 total aligned reads displayed.  

`TAGGCTGGACTTGGACAAGACAGACTGGCTGGAAGAAACCAGAAGTTACCAAGCTGAGTCATAAGGTGTTCGATAAGGACACTATGACCCACATCAAGGCTGGTGATTGGGTTAAGGTTGACATGGGAGTTGTTGGTGGATACGGCTACGTCCGCTCAGTTAGTGGCAAATATGCACAAGTGTCATACATCACAGGTGTTACTCCACGCGGTGCAATCGTTGCCGATAAGACCAACATGATTCACACAGGTTTCTTGACAGTTGTTTCATATGAAGAGATTGTTAAGTCACGATAATCAATAGGAGAAATCAATATGATCGTTTCTGACATCGAAGCTAACGCCCTCTTAGAGAGCGTCACTAAGTTCCACTGCGGGGTTATCTACGACTACTCCACCGCTGAGTACGTAAGCTACCGTCCGAGTGACTTCGGTGCGTATCTGGATGCGCTGGAAGCCGAGGTTGCACGAGGCGGTCTTATTGTGTTCCACAACGGTCACAAGTATGACGTTCCTGCATTGACCAAACTGGCAAAGTTGCAATTGAACCGAGAGTTCCACCTTCCTCGTGAGAACTGTATTGACACCCTTGTGTTGTCACG  >  NC_001604.1/14039‑14639`  

                                                                                                                                                                                                                                                                                                            `|`                                                                                                                                                                                                                                                                                                              
`ccggagatgtgtagaagagacatactacgagaagtttcgctggagagtcccatgctaatacgactcactaaaggagacacaccatgttcaaactgattaagaagttaggccaactgctggttcgtatgtacaacgtggaagccaagcgactgaacgatgaggctcgtaaagaggccacacagtcacgcgctctggcgattcgctccaacgaactggctgacagtgcatccactaaagttaccgaggctgcccgtgtggcaaaccaagctcaacagctttccaaattctttgagtaatcaat                                                                                                                                                                                                                                                                                                              <  1:315672‑M2/1‑1 (MQ=255)`  
`acagcttgatgactgtccagacgactacgagaagtttcgctggagagtcccattctaatacgactcactaaaggagacacaccatgttcaaactgattaagaagttaggccaactgctggttcgtatgtacaacgtggaagccaagcgactgaacgatgaggctcgtaaagaggccacacagtcacgcgctctggcgattcgctccaacgaactggctgacagtgcatccactaaagttacctaggctgcccgtgtggcaaaccaagctcaacagctttccaaattctttgagtaatcaat                                                                                                                                                                                                                                                                                                              <  1:164997‑M2/1‑1 (MQ=255)`  
  `ttcttgatgactttccagacgactacgagaagtttcgctggagagtcccattctaatacgactcactaaaggagacacaccatgttcaaactgattaagaagttaggccaactgctggttcgtatgtacaacgtggaagccaagcgactgaacgatgaggctcgtaaagaggccacacagtcacgcgctctggcgattcgctccaacgaactggctgacagtgcatccactaaagttaccgaggctgcccgtgtggcaaaccaagctcaacagctttccaaattctttgagtaatcaaTAg                                                                                                                                                                                                                                                                                                            <  1:419588‑M2/3‑1 (MQ=255)`  
   `tcttgattactttccagacgactacgagaagtttcgctggagagtcccattctaatacgactcactaaaggagacacaccatgttcaaactgattaagaagttaggccaactgctggttcgtatgtacaacgtggaagccaagcgactgaacgatgaggctcgtaaagaggccacacagtcacgcactctggcgattcgctccaacgaactggctgacagtgcatccactaaagttaccgaggctgcccgtgtggcaaaccaagctcaacagctttccaaattctttgagtaatcaaTAgg                                                                                                                                                                                                                                                                                                           <  1:110685‑M2/4‑1 (MQ=255)`  
   `tcttgatgactttccagacgactacgctaagtttcgctggagagtcccattctaatacgactcactaaaggagacacaccatgttcaaactgattaagaagttaggccaactgctggttcgtatgtacaacgtggaagccaagcgactgaacgatgaggctcgtaaagaggccacacagtcacgcgctctggcgattcgctccaacgaactggctgacagtgcatccactaaagttaccgaggctgcccgtgtggcaaaccaagctcaacagctttccaaattctttgagtaatcaaTAgg                                                                                                                                                                                                                                                                                                           <  1:311041‑M2/4‑1 (MQ=255)`  
   `tcttgatgactttccagacgactacgagaagtttcgctggagagtcacattctaatactactcactaaaggagacacaccatgttcaaactgattaagaagttaggccaactgctggttcgtatgtacaacgtggaagccaagcgactgaacgatgaggctcgtaaagaggccacacagtcacgcgctctggcgattcgctccaacgaactggctgacagtgcatccactaaagttaccgaggctgcccgtgtggcaaaccaagctcaacagctttccaaattctttgagtaatcaaTAgg                                                                                                                                                                                                                                                                                                           <  1:395478‑M2/4‑1 (MQ=255)`  
   `tcttgatgactgtccagacgactacgagaagtttcgctggagagtcccattctaatacgactcactaaaggagacacaccatgttcaaactgattaagaagttaggccaactgctggttcgtatgtacaacgtggaagccaagcgactgaacgatgaggctcgtaaagaggccacacagtcacgcgctctggcgattcgctccaacgaactggctgacagtgcatccactaaagttaccgaggctgcccgtgtggcaaaccaagctcaacagctttccaaattctttgagtaatcaaTAgg                                                                                                                                                                                                                                                                                                           <  1:369735‑M2/4‑1 (MQ=255)`  
    `cttgatgactttccagacgactacgagaagtttcgctggagagtcccattctaatacgactcactaaaggagacacaccatgttcaaactgattaagaagttaggccaactgctggttcgtatgtacaacgtggaagccaagcgactgaacgatgaggctcgtaaagaggccacacagtcacgcgctctggcgattcgctccaacgaactggctgacagtgcatccactaaagttaccgaggctgcccgtgtggcaaaccaagctcaacagctttccacattctttgagtaatcaaTAGga                                                                                                                                                                                                                                                                                                          >  1:135047‑M2/297‑301 (MQ=255)`  
    `cttgatgactttccagacgactacgagaagtttcgctggagagtcccattctaatacgactcactaaaggagacacaccatgttcaaactgattaagaagttaggccaactgctggttcgtatgtacaacgtggaagccaagcgactgaacgacgaggctcgtaacgaggccacacagtcacgcgctctggcgattcgctccaacgaactggctgacagtgcatccaccaaagttaccgaggctacccgtgtggcaaaccaagcccaacagctttccaaattctttgagtaatcaaTAGga                                                                                                                                                                                                                                                                                                          >  1:477227‑M2/297‑301 (MQ=255)`  
          `gactttccatacgactacgagaagtttcgctggagagtcccattctaagacgactcactaaaggagacacaccaggttcaaactgattaagaaggtaggccaactgctggttcgtatgtacaacgtggaatccaagcgactgaacgacgaggctcgtaaagaggccacacagtcacgcgctctggcgattcgctccaacgaactggctgacagtgcatccactaaagttaccgaggctgaccatgtggcaaaccaagctcagcagctttccaaattctttgagtaatcaaTAGGAGAAATc                                                                                                                                                                                                                                                                                                    <  1:35986‑M2/11‑1 (MQ=255)`  
          `gactttccagacgactacgagaagtttcgctggagagtcccattctaatacgactcactaaaggagacacaccatgttcaaactgattaagaagttaggccaactgctggttcgtatgtacaacgtggaagccaagcgactgaacgatgaggctcgtaaagaggccacacagtcacgcgctctggcgattcgctccaacgaactggctgacagtgcatccactaaagttaccgaggctgcccgtgtggcaaaccaagctcaacagctttccaaattctttgagtaatcaaTAGGAGAAATc                                                                                                                                                                                                                                                                                                    <  1:208716‑M2/11‑1 (MQ=255)`  
                    `actacgagaagtttcgctggagagtcccattctaatacgactcacactaaaggagacacaccatgttcaaactgattaagaagttaggccaactgctggttcgtatgtacaacgtggaagccaagcgactgaacgatgaggctcgtaaagaggccacacagtcacgcgctctggcgattcgctccaacgaactggctgacagtgcatccactaaagttaccgaggctgcccgtgtggcaaaccaagctcaacagctttccaaattctttgagtaatcaaaCAGGAGAAATCAATATGATCg                                                                                                                                                                                                                                                                                          <  1:148950‑M2/21‑1 (MQ=255)`  
                     `cgactacgagaagtttcgctggagagtcccattctaatacgactcactaaaggagacacaccatgttcaaactgattaagaagttaggccaactgctggttcgtatgtacaacgtggaagccaagcgactgaacgatgaggctcgtaaagaggccacacagtcacgcgctctggcgattcgctccaacgaactggctgacagttcatccactaaagttaccgaggctgcccgtgtggcaaaccaagctcaacagctttccaaattctttgcgtaatcaataggctgtctcttatacacatc                                                                                                                                                                                                                                                                                         >  1:333728‑M2/280‑283 (MQ=255)`  
                       `ctacgagaagtttcgctggagagtcccattctaatacgactcactaaaggagacacaccatgttcaaactgattaagaagttaggccaactgctggttcgtatgtacaacgtggaagccaagcgactgaacgatgaggctcgtaaagaggccacacagtcacgcgctctggcgattcgctccaacgaactggctgacagtgcatccactaaagttaccgaggctgcccgtgtggcaaaccaagctcaacagctttccaaattctttgagtaatcaaaCAGGAGAAATCAATATGATCGttt                                                                                                                                                                                                                                                                                       >  1:100160‑M2/278‑301 (MQ=255)`  
                                                   `ttctaatacgactcactaaaggagacacaccatgttcaaactgattaagaagttaggccaactgctggttcgtatgtacaacgtggaagccaagcgactgaacgatgaggctcgtaaagaggccacacagtcacgcgctctggcgattcgctccaacgaactggctgacagtgcatccactacagttaccgaggctgcccgtgtggccaacaaagctcaacagctttccaaattctttgagtaatcaaaTAGGAGAACTCAATATGATCGTTTCTGACATCGACGCTAACGCCCGCTTaga                                                                                                                                                                                                                                                           >  1:389573‑M2/250‑301 (MQ=255)`  
                                                   `ttctaatacgactcactaaaggagacacaccatgttcaaactgattaagaagttaggccaactgctggttcgtatgtacaacgtggaagccaagcgactgaacgatgaggctcgtaaagaggccacacagtcacgcgctctggcgattcgctccaacgaactggctgacagtgcatccactaaagttaccgaggctgcccgtgtggcaaaccaagctcaacagctttccaaattctttgagtaatcaaaCAGGAGAAATCAATATGATCGTTTCTGACATCGAAGCTAAAGCCCTCTTaga                                                                                                                                                                                                                                                           >  1:129789‑M2/250‑301 (MQ=255)`  
                                                    `ttctaatacggctcactaaaggagacacagcatgtacaaaaggattaagaagtgaggcaaactgctggttcgtatgtaaaacgtgaaagccaagagaatgaacgatgaggctcgtaaagaggccacacagtcacgcgctctggagattcgatccaacgaactggctgaaagtgcatccactaaagttaccgaggctgcccgtgtggcaaaccaagctcaacagctttccaaattctgtgagtaatcaaTAGGAGAAATCAATATGATCGTTTCTGACATCGAAGCTAACGCCCTCTTagag                                                                                                                                                                                                                                                          <  1:660417‑M2/53‑1 (MQ=255)`  
                                                    `ttctaatacgaatcactaaaggagacacaccatgttcaaactgattaagaagttaggcaaactgatggttcgtatgtacaacgtggaagccaagcgactgaacgatgaggctcgtaaagaggccacacagtcacgcgctctggcgattcgctccaacgaactggctgacagtgcatccactaaagttaccgaggctgcccgtgtggcaaaccaagctcaacagctttccaaattctttgagtaatcaaTAGGAGAAATCAATATGATCGTTTCTGACATCGAAGCTAACGCCCTCTTagag                                                                                                                                                                                                                                                          <  1:353303‑M2/53‑1 (MQ=255)`  
                                                    `tctaatacgactcactaaaggagacacaccatgttcaaactgattaagaagttaggccaactgctggttcgtatgtacaacgtggaagccaagcgactgaacgattaggctcgtaaagaggccacacagtcacgcgctctggcgattcgctccaacgaactggctgacagtgcatccactaaagttaccgaggctgcccgtgtggcaaaccaagctcaacagctttccaaattctttgagtaatcaaaCAGGAGAAATCAATATGATCGTTTCTGACATCGAAGCTAACGCCCTCTTagag                                                                                                                                                                                                                                                          <  1:340318‑M2/53‑1 (MQ=255)`  
                                                    `tataagagacagcactaaaggagacacaccatgttcaaactgattaagaagttaggccaactgctggttcgtatgtacaacgtggaagccaagcgactgaacgatgaggctcgtaaagaggccacacagtcacgcgctctggcgattcgctccaacgaactggctgacagtgcatccactaaagttaccgaggctgcccgtgtggcaaaccaagctcaacagctttccaaattctttgagtaatcaaaCAGGAGAAATCAATATGATCGTTTCTGACATCGAAGCTAACGCCCTCTTagag                                                                                                                                                                                                                                                          <  1:592128‑M2/53‑1 (MQ=255)`  
                                                    `gtgggctcggagatgtgtataagagacagccatgttcaaactgattaagaagttaggccaactgctggttcgtatgtacaacgtggaagccaagcgactgaacgatgaggctcgtaaagaggccacacagtcacgcactctggcgattcgctccaacgaactggctgacagtgcatccactaaagttaccgaggctgcccgtgtggcaaaccaagctcaacagctttccaaattctttgagtaatcaaTAGGAGAAATCAATATGATCGTTTCTGACATCGAAGCTAACGCCCTCTTagag                                                                                                                                                                                                                                                          <  1:569445‑M2/53‑1 (MQ=255)`  
                                                    `ggagatgtgtataagagacaggagacacaccatgttcaaattgattaagaagttaggccaactgctggttcgtatgtacaacggggaagccaagcgactgaacgatgaggctcgtaaagaggccacacagtcacgcgctctggcgattcgctccaacgaactggctgacagtgcatccactaaagttaccgaggctgcccgtgtggcaaaccaagctcaacagctttccaaattctttgagtaatcaaTAGGAGAAATCAATATGATCGTTTCTGACATCGAAGCTAACGCCCTCTTagag                                                                                                                                                                                                                                                          <  1:620632‑M2/53‑1 (MQ=255)`  
                                                        `aatacgactcactaaaggagacacaccatgttcaaactgattaagaagttaggccaactgctggttcgtatgtacaacgtggaagccaagcgactgaacgatgaggctcgtaaagcggccacacagtcacgcgctctggcgattcgctccaacgaactggctgacagtgcatccactaaagttaccgcggctgcccgtgtggcaaaccaagctcaacagctttccaaattctttgagtaatcaaTAGGAGAAATCAATATGATCGTTTCTGACATCGAAGCTAACGCCACCTTAGAGGGCg                                                                                                                                                                                                                                                      >  1:429826‑M2/245‑301 (MQ=255)`  
                                                            `gactcactaaaggagacacaccatattcaaactgattaagaagttaggccaactgctggttcgtatgtacaacgtggaagccaagcgactgaacgatgaggctcgtaaagaggccacacagtcacgcgctctggcgattcgctccaacgaactggctgacagtgcatccactaaagttaccgaggctgcccgtgtggcaaaccaagctcaacagctttcaaaattctttgagtaatcaaaCAGGAGAAATCAATATGATCGTTTCTGACATCGAAGCTAACGCCCTCTTAGAGAGCGTCAc                                                                                                                                                                                                                                                  <  1:273239‑M2/61‑1 (MQ=255)`  
                                                             `actcactaaaggagacacaccatgttcaaactgattaagaagttaggccaactgctggttcgtatgtacaacgtggaagccaagcgactgaacgatgaggctcgtaaagaggccacacagtcacgcgctctggcgattcgctccaacgaactggctgacagtgcatccactaaagttaccgaggctgcccgtgtggcaaaccaagctcaacagctttccaaattctttgagtaatcaaaCAGGAGAAATCAATATGATCGTTTCTGACATCGAAGCTAACGCCCTCTTAGAGAGCGTCACt                                                                                                                                                                                                                                                 >  1:400610‑M2/240‑301 (MQ=255)`  
                                                                 `cactaaaggagacacaccatgttcaaactgattaagaagttaggccaactgctggttcgtatgtacaacgtggaagccaagcgactgaacgatgaggctcgtaaagaggccacacagtcacgcgctctggcgattcgctcccacgaactggctgacagtgcatacactaaagttaccgaggctgcccgtgtggcaaaccaagctcaacagctttacaaattctttgagtaatcaaTAGGAGAAATCAATATGATCGTTTCTGACATCGAAGCTAACGCCCTCTTCGAGAGCGCCACTAAGt                                                                                                                                                                                                                                             >  1:115880‑M2/236‑301 (MQ=255)`  
                                                                 `cactaaaggagacacaccatgttcaaactgattaagaagttaggccaactgctgattcgtatgtccaacgtggaagccatgctattgaacgatgagtctcgttaagaggccacacagtcacgcgctctggcgcttcggacgaacgaactggcagacagtgcatctactaacgttcccgaggctgcccgtgtggcaaacaaagctcaacagctttccaacttctttgagtaatcaaTAGGAGAACTCAATATGAGTGTTGCTGACAtcgaaactaaccccctcttcaacttccctctttacc                                                                                                                                                                                                                                             >  1:363953‑M2/236‑284 (MQ=255)`  
                                                                   `aaaaggagacaccccatgttcaaactgattaagaagttaggccaactgctggttcgtatgtacaacgtggaagccaagcgactgaacgatgaggctcgtaaagaggccacacagtcacgcgctctggcgattcgctccaacgaactggctgacagtgcatccactaaagttaccgaggctgcccgtgtggcaaaccaagctcaacagctttccaaattctttgagtaatcaaaCAGGAGAAATCAATATGATCGTTTCTGACATCGAAGCTAACGCCCTCTTAGAGAGCGTCACTAAGTTc                                                                                                                                                                                                                                           <  1:122983‑M2/68‑1 (MQ=255)`  
                                                                     `aaaggagacacaccatgttcaaactgattaagaagttaggccaactgctggttcgtatgtacaacgtggaagccaagcgactgaacgatgaggctcgtaaagaggccacacagtcacgcgctctggcgattcgctccaacgaactggctgacagtgcatccactaaagttaccgaggctgcccgtgtggcaaaccaagctcaacagctttccaaattctttgagtaatcaaTAGGAGAAATAAATATGATCGTTTCTGACATCGAAGCTAACGCCCTCTTAGCGAGCGTCACTAAGTTCCa                                                                                                                                                                                                                                         >  1:537029‑M2/232‑301 (MQ=255)`  
                                                                        `ggagacacaccatgttcaaactgattaagaagttatgccaactgctggttcgtatgtacaacgtggaagccaagcgactgaacgatgaggctcgtaaagaggccacacagtcacgcgctctggcgattagctccaacgaactggctgacagtgcatccactaaagttaacgaggcttcccgtgtggcaaaccaagctcaacagctttccaaattctttgagtaatcaaTATGAGAAATCAATATGCTCGTTTCTGACATCGACGCTAACGCCCTCTTAGACAGCGTACATAAGTTCCActt                                                                                                                                                                                                                                      >  1:666930‑M2/229‑300 (MQ=255)`  
                                                                        `caggacacaccatgttcaaactgattaagaagttaggccaactgctggttcgtatgtacaacgtggaagccaagcgactgaacgatgaggctcgtaaagaggccacacagtcacgcgctctggcgattcgctccaacgaactggctgacagtgcatccactaaagttaccgaggctgcccgtgtggcaaaccaagctcaacagctttccaaattctttgagtaatcaaTAGGAGAAATCAATATGATCGTTTCTGACATCGAAGCTAACGCCCTCTTAGAGAGCGTCACTAAGTTCCACTg                                                                                                                                                                                                                                      <  1:483958‑M2/73‑1 (MQ=255)`  
                                                                             `aacaccatgatcaaactgagtaagaagttaggccaactgctggttcgtatgtacaacgtggaagccaagcgactgaacgatgaggctcgtaaagaggccacacagtcacgcgctctggcgattcgctccaacgaactggctgacagtgcatccactaaagttaccgaggctgcccgtgtggcaaaccaagctcaacagctttccaaattctttgagtaatcaaTAGGAGAAATCAATATGATCGTTTCTGACATCGAAGCTAACGCCCTCTTAGAGAGCGTCACTAAGTTCCACTGCgggg                                                                                                                                                                                                                                 <  1:654009‑M2/78‑1 (MQ=255)`  
                                                                                        `caaactgatgaagaagttaggccaactgctggttcgtatgtacaacgtggaagccaagcgactgaacgatgaggctcgtaaagaggccacacagtcacgcgctctggcgattcgctccaacgaactggctgacagtgcatccactaaagttaccgaggctgcccgtgtggcaaaccaagctcaacagctttccaaattctttgagtaatcaaTAGGAGAAATCAATATGATCGTTTCTGACATCGAAGCTAACGCCCTCTTAGAGAGCGTCACTAAGTTCCACTGCGGGGTTATCTACGac                                                                                                                                                                                                                      <  1:261155‑M2/89‑1 (MQ=255)`  
                                                                                        `aaactgattaagaaattaggccaactgctggttcgtatctacaacgtggaagccaatcgactgaacgatgaggctcgtaaagaggccacacagtcacgcgctctggcgattcgctccaacgaactggctgacagtgcatccactaaagttaccgaggctgcccgtgtggcaaaccaagctcaacagctttccaaattctttgagtaatcaaaCAGGAGAAATCAATATGATCGTTTCTGACATCGAAGCTAACGCCCTCTTAGAGAGCGTCACTAAGTTCCACTGCGGGGTTATCTACGac                                                                                                                                                                                                                      <  1:501586‑M2/89‑1 (MQ=255)`  
                                                                                         `gacaggattaagaagttaggccaactgctggttcgtatgtacaacgtggaagccaagcgactgaacgatgaggctcgtaaagaggccacacagtcacgcgctctggcgattcgctccaacgaactggctgacagtgcatccactaaagttaccgaggctgcccgtgtggaaaaccaagctcaacagctttccaaattctttgagtaatcaaTAGGAGAAATCAATATGATCGTTTCTGACATCGAAGCTAACGCCCTCTTAGAGAGCGTCACTAAGTTCCACTGAGGGGTTATCTACGact                                                                                                                                                                                                                     <  1:154744‑M2/90‑1 (MQ=255)`  
                                                                                         `aaatgattaagaagttagggcaactgctggttcggatgtaaaacgtggaagccaagcgactgaaagatgaggctcgtaaagaggacataaagtcacgcgcgcgggcgattcgctccaacgaactggatgacagtgcatccactaaagttaccgaggctgcccgtgtggcaaaccaagctcaacagctgtccaaattctttgagtaatcaaaCAGGAGAAATCAATATGATCGTTTCTGACATCGAAGCTAACGCCCTCTTAGAGAGCGTCACTAAGTTCCACTGCGGGGTTATCTACGact                                                                                                                                                                                                                     <  1:203231‑M2/90‑1 (MQ=255)`  
                                                                                              `gattaagaagttaggccaactgctggttcgtatgtacaacgtggaagccaagcgactgaacgatgaggctcgtaaagaggccacacagtcacgcgctctggcgattcgctccaacgaactggctgacagtgcatccactaaagttaccgaggctgcccgtgtggcaaaccaagctcaacagctttccaaattctttgagtaagcaaTAGGAGAAATCAATATGATCGTTTCTGACATCGACGCTAACGCCCTCTGAGAGAGCGCCACTAAGTTCCGAGGAGGGGGTAgctacgacatgtga                                                                                                                                                                                                                >  1:631193‑M2/207‑295 (MQ=255)`  
                                                                                                   `tgtataagagccagctgctggttcgtatggacaacgtggaagccaagcgactgaacgatgaggctcgtaaagaggccacacagtcacgcgctctggcgattcgctccaacgaactggctgacagagcatccactaaagttaccgaggctgcccgtgtggcaaaccaagctcaacagctttccaaattctttgagtaatcaaTAGGAGAAATCAATATGATCGTTTCTGACATCGAAGCTAACGCCCTCTTAGAGAGCGTCACTAAGTTCCACTGCGGGGTTATCTACGACTACTCCACCGc                                                                                                                                                                                                           <  1:531031‑M2/100‑1 (MQ=255)`  
                                                                                                        `ttaggccaacgtctggttcgtatgtacaacgtgtaagccaagcgactgaactatgaggctcgtaaagaggccacacagtcacgcgctctgtcgattcgctccaacgaactggctgacagtgcatccactaaagttaccgaggctgcccgtgtggcaaaccaagctcaacagctttccaaattctttgagtaatcaaTAGGAGAAATCAATATGATCGTTTCTGACATCGAAGCTAACGCCCTCTTAGAGAGCGTCACTAAGTTCCACTGCGGGGTTATATACGACTACTCCACCGCTGAGt                                                                                                                                                                                                      <  1:220393‑M2/105‑1 (MQ=255)`  
                                                                                                             `ccaactgctggttcgtatgtacaacgtggaagccaagcgactgaacgatgaggctcgtaaagaggccacacagtcacgcgctctggcgattcgctccaacgaactggctgacagtgcatccactaaagttaccgaggctgcccgtgtggcaaaccaagctcaacagctttccaaattctttgagtaatcaaTAGGAGAAATCAATATGATCGTTTCTGACATCGAAGCTAACGCCCTCTTAGAGAGCGTCACTAAGTTCCACTGCGGGGTTATCTACGACTACTCCACCGCTGAGTACGTa                                                                                                                                                                                                 <  1:86175‑M2/110‑1 (MQ=255)`  
                                                                                                             `caactgctggttcgtatgtacaacgtggaagccaagcgactgaacgatgaggctcgtaaagaggccacacagtcacgcgctctggcgattcgctccaacgaactggctgacagtgcatccactaaagttaccgaggctgcccgtgtggcaaaccaagctcaacagctttccaaattctttgagtaatctaaCAGGAGAAATCAATATGATCGTTTCTGACATCGAAGCTAACGCCCTCTTAGAGAGCGTCACTAAGTTCCACTGCGGGGTTATCTACGACTACTCCACCGCTGAGTACGTa                                                                                                                                                                                                 <  1:214936‑M2/110‑1 (MQ=255)`  
                                                                                                               `actgctggttcgtatgtacaacgtggaagccaagcgactgaacgatgaggctcgtaaagaggccacacagtcacgcgctctggcgattcgctccaacgaactggctgacagtgcatccactaaagttaccgaggctgcccgtgtggcaaaccaagctcaacagctttccaaattctttgagtaatcaaaCAGGAGAAATCAATATGATCGTTTCTGACATCGAAGCTAACGCCCTCTTAGAGAGCGTCACTACGTTCCACTGCGGGGTTATCTACGACTACTCCACCGCTGAGTACGTAAg                                                                                                                                                                                               >  1:602133‑M2/190‑301 (MQ=255)`  
                                                                                                               `actgctggttcgtatgtacaacgtggaagccaagcgactgaacgatgaggctcgtaaagaggccacacagtcacgcgctctggcgattcgctccaacgaactggctgacagtgcatccactaaagttaccgaggatgcccgtgtggcaaaccaagctcaacagctttccaaattctttgagtaataaaaCAGGAGAAATCAATATGATCGTTTCTGACATCGAAGCTAACGCCCTCTTAGAGAGCGTCACTAAGTTCCACTGCGGGGTTATCTACGACTACTCCACCGCTGAGTACGTAAg                                                                                                                                                                                               <  1:626334‑M2/112‑1 (MQ=255)`  
                                                                                                                `ctgctggttcgtatgtacaacgtggaagccaagcgactgaacgatgaggctcgtaaagaggccacacagtcacgcgctctggcgattcgctccaacgaactggctgacagtgaatccactaaagttaccgaggctgcccgtgtggcaaaccaagctcaacagctttccaaattctttgagtaatcaaaCAGGAGAAATCAATATGATCGTTTCTGACATCGAAGCTAACGCCCTCTTAGAGAGCGTCACTAAGTTCCACTGCGGGGTTATCTACGACTACTCcaccgctgagctgtctctt                                                                                                                                                                                              >  1:172002‑M2/189‑292 (MQ=255)`  
                                                                                                                    `tggttcgtatgtacaacgtggaagccaagcgactgaacgatgaggctcgtaaagaggccacacagtcacgcgctctggcgattcgctccaacgaactggctgacagtgcatccactaaagttaccgaggctgcccgggtggcaaatcaagctcaacagctttccaaattctttgagtaatcaaaCAGGAGAAACCATTATGATCGTTTCTGTCATCGAAGCTAAAGCCCTCTTAGAGCGCGTCACTAAGTTCCACTGCGGGGGTATCTACGACTACTCCACCGCTGAGTACGTAAGCTAcc                                                                                                                                                                                          >  1:636481‑M2/185‑301 (MQ=255)`  
                                                                                                                         `cgtatgtacaacgtggaagccaagcgactgaactatgaggctcgtaaagaggccacacagtcactcgctctggcgattctctccaacgaactggctgacagtgcatccactaaagttaccgaggctgcccgtgtggcaaaccaagctcaacagctttccaaattctttgagtaatcaaaCAGGAGAAATCAATCTGATCGTTTCTGACATCGAAGCTAACGCCCTCTTAGAGAGCGTCACTCAGTTCCACTGCGGGGTTATCTACGACTACTCCACCGCTGAGTACGTAAGCTACCGTCCg                                                                                                                                                                                     >  1:58543‑M2/180‑301 (MQ=255)`  
                                                                                                                         `cgtatgtacaacgtggaagccaagcgactgaacgatgaggctcgtaaagaggccacacagtcacgcgctctggcgattcgctccaacgaactgtctgacagtgcatccactaaagttacctagtctgcccgtgtggcaaaccaagctcaacagctttccaaattctttgagtaatcaaaAAGGTGAAATCAATCTGATCGTTTCTAACATCGAAGATAACTCCCTCTTTTAGAGCGTCACTAAGTTCCACTGCGGGGTTATCTACGACTACTCTAATGATGAGTACGTAAGCTCCCGTCCg                                                                                                                                                                                     >  1:380993‑M2/180‑301 (MQ=255)`  
                                                                                                                            `tatgtacaacgtggaagccaagcgactgaacgatgaggctcgtaaagaggccacacagtcacgcgctctggcgattcgctccaacgaactggctgacagtgcatccactaaagttaccgaggctgcccgtgtggcaaaccaagctcaacagctttccaaattctttgagtaatcaaTAGGAGGAATCAATATGATCGTTTCTGACATCGAAGCTAACGCCCTCTTAGAGAGCGTCACTAAGTTTCACTGCGGGGTTATTTACGACTACTTCACCGCCGAGTACGTAAGCTACCGTCCGagg                                                                                                                                                                                  >  1:558195‑M2/177‑300 (MQ=255)`  
                                                                                                                              `gtacaacgtggaagccaagcgactgaacgatgaggctcgtaaagaggccacacagtcacgcgctctggcgattcgctccaacgaactggctgacagtgcatccactaaagttaccgaggctgcccgtgtggcaaacccagctcaacagctttccaaattctttgagtaatcaaaCAGGAGAAATCAATATGATCGTTTCTGACATCGAAGCTAACGCCCTCTTAGAGAGCGTCACTAAGTTCCACTGCGGGGTTATCTACGACTACTCCACCGCTGAGTACGTAAGCTACCGTCCGAGTGa                                                                                                                                                                                >  1:298035‑M2/175‑301 (MQ=255)`  
                                                                                                                                     `gtggaagccaagcgactgaacgatgaggctcgtaaagaggccacacagtcacgcgctctggcgattcgctccaacgaactggctgacagtgcatccactaaagttaccgaggctgcccgtgtggcaaaccaagctcaacagctttccaaattctttgagtaatcaaaCAGGAGAAATCAATATGATCGTTTCTGACATCGAAGCTAACGCCCTCTTAGAGAGCGTCACTAAGTTCCACTGCGGGGTTATCTACGACTACTCCACCGCTGAGTACGTAAGCTACCGTCCGAGTGACTTCGGt                                                                                                                                                                         <  1:516344‑M2/134‑1 (MQ=255)`  
                                                                                                                                     `gtggaagccaagcgactgaacgatgaggctcgtaaagaggccacacagtcacgcgctctggcgattcgctccaacgaactggctgacagtgcatccactaaagttaccgaggctgcccgtgtggcaaaccaagctcaacagctttccaaattctttgagtaatcaaaCAGGAGAAATCAATATGATCGTTTCTGACATCGAAGCTAACGCCCTCTTAGAGAGCGTCACTAAGTTCCACTGCGGGGTTATCTACGACTACTCCACCGCTGAGTACGTAAGCTACCGTCCGAGTGACTTCGGt                                                                                                                                                                         >  1:159920‑M2/168‑301 (MQ=255)`  
                                                                                                                                      `gtggaagccaagcgactgaacgatgaggctcgtaaagaggccacacagtcacgcgctctggctattcgctccaacgaactggctgacagtgcatccactaaagttaccgaggctgcccgtgtggcaaaccaagctcaacagctttccaaattctttgagtaatcaaTAGGAGAAATCAATATGATCGTTTCTGACATCGAAGCTAACGCCCTCTTAGAGAGCGTCACTAAGTTCCACTGCGGGGTTATCTACGACTACTCCACCGCTGAGTACGTAAGCTACCGTCCGAGTGACTTCGGTg                                                                                                                                                                        <  1:577828‑M2/135‑1 (MQ=255)`  
                                                                                                                                      `gtggaagccaagcgactgaacgatgaggctcgtaaagaggccacacagtcacgcgctctggcgattcgctccaacgaactggctgacagtgcatccactaaagttaccgaggctgcccgtgtggcaaaccaagctcaacagctttccaaattctttgagtaatcaaTAGGAGAAATCAATATGATCGTTTCTGACATCGAAGCTAACGCCCTCTTAGAGAGCGTCACTAAGTTCCACTGCGGGGTTATATACGACTACTCCACCGCTGAGTACGTAAGCTACCGTCCGAGTGACTTCGGTg                                                                                                                                                                        >  1:520753‑M2/167‑301 (MQ=255)`  
                                                                                                                                      `gtggaagccaagcgactaaacgatgaggctcgtaaagaggccacacaggcacgcgctctggcgattcgctcaaacgaactggctgacagtgcatccactaaagttaccgaggctgcccgtgtggcaaaccaagctcaacagctttccaaattctttgagtaatcaaTAGGAGAAATCAATATGATCGTTTCTGACATCGAAGCTAACGCCCTCTTAGAGAGCGTCACTAAGTTCCACTGCGGGGTTATCTACGACTACTCCACCGCTGAGTACGTAAGCTACCGTCCGAGTGACTTCGGTg                                                                                                                                                                        <  1:23676‑M2/135‑1 (MQ=255)`  
                                                                                                                                             `ccaagcgactgaacgatgaggctcgtaaagaggccacacagtcacgcgctctggcgattcgctccaacgaactggctgacagtgcatccactaaagttaccgaggctgcccgtgtggcaaaccaagctcaacagctttccaaattctttgagtaatcaaTAGGAGAAATCAATATGATCGTTTCTGACATCGAAGCTAACGCCCTCTTAGAGAGCGTCACTAAGTTCCACTGCGGGGTTATCTACGACTACTCCACCGCTGAGTACGTAAGCTACCGTccgagtgacttcctgtctcttat                                                                                                                                                                 >  1:407453‑M2/160‑290 (MQ=255)`  
                                                                                                                                              `aagcgactgaacgatgaggctcgtaaagaggccacacagtcacgcgctctggcgattcgctcaaacgaactggctgacagtgcatccactaaagttaccgaggctgcccgtgtggcaaaccaagctcaacagctttccaaattctttgagtaatcaaaCAGGAGAAATCAATATGATCGTTTCTGACATCGAAGCTAACGCCCTCTTAGAGAGCGTCACTAAGTTCCACTGCGGGGTTATCTACGACTACTCCACCGCTGAGTACGTAAGCTACCGTCCGAGTGACTTCGGTGCGTATCTg                                                                                                                                                                <  1:424616‑M2/143‑1 (MQ=255)`  
                                                                                                                                                `agcgactgaacgatgaggctcgtaaagaggccacacagtcacgcgctctggcgattcgctccaacgaactggctgacagtgcatccactaaagttaccgaggctgcccgtgtggcaaaccaagctcaacagctttccaaattctttgagtaatcaaTAGGAGAAATCAATATGATCGTTTCTGACATCGAAGCTAACGCCCTCTTAGAGAGCGTCACTAAGTTCCACTGCGGGGTTATCTACGACTACTCCACCGCTGAGTACGTAAGCTACCGTCCGAGTGACTTCGGTGCGTATCTGGa                                                                                                                                                              <  1:249136‑M2/145‑1 (MQ=255)`  
                                                                                                                                                `agcgactgaacgatgaggctcgtaaagaggccacacagtcacgcgctctggcgattcgctccaacgaactggctgacagtgcatccactaaagttaccgaggctgcccgtgtggcaaaccaagctcaacagctttccaaattctttgagtaatcaaTAGGAGAAATCAATATGATCGTTTCTGACATCGAAGCTAACGCCCTCTTAGAGAGCGTCACTAAGTTCCACTGCGGGGTTATCTACGACTACTCCACCGCTGAGTACGTAAGCTACCGTCCGAGTGACTTCGGTGCGTATCTGGa                                                                                                                                                              <  1:453786‑M2/145‑1 (MQ=255)`  
                                                                                                                                                       `gaacgatgaggctcgtaaagaggccacacagtcacgcgctctggcgattcgctccaacgaactggctgacagtgcatccactaaagttaccgaggctgcccgtgtggcaaaccaagctcaacagctttccaaattctttgagtaatcaaTAGGAGAAATCAATATGATCGTTTCTGACATCGAAGCTAACGCCCTCTTAGAGAGCGTCACTAAGTTCCACTGCGGGGTTATCTACGACTACTCCACCGCTGAGTACGTAAGCTACCGTCCGAGTGACTTCGGTGCGTATCTGGATGCGCTg                                                                                                                                                       >  1:287102‑M2/150‑301 (MQ=255)`  
                                                                                                                                                                `gggcacttaacgtggccacaccgccacccgctctggcgtttcgcgccaacgaacgggctgacagtgcatccactaaagttaccgagtctgcccgtttggcaaccaagctcaacagctttccaaattctttgagtaatcaaTAGGAGAAATCAATATGATCGTTTCTGACATCGAAGCTAACGCCCTCTTAGAGAGCGTCACTAAGTTCCACTGCGGGGTTATCTACGACTACTCCACCGCTGAGTACGTAAGCTACCGTCCGAGTGACTTCGGTGCGTATCTGGATGCGCTGGAAGCCGAg                                                                                                                                              <  1:56313‑M2/161‑1 (MQ=255)`  
                                                                                                                                                                   `cgtaaagaggccactcagtcacgcgctctggcgattcgctccaacgaactggctgacagtgcatccactaaagttaccgaggctgcccgtgtggcaaaccaagctcaacagctttccaaattctttgagtaatcaaaCAGGAGAAATCAATATGATCGTTTCTGACATCGAAGCTAACGCCCTCTTAGAGAGCGTCACTAAGTTCCACTGCGGGGTTATCTACGACTACTCCACCGCTGAGTACGTAAGCTACCGTCCGAGTGACTTCGGTGCGTATCTGGATGCGCTGGAAgccgactgt                                                                                                                                           >  1:649020‑M2/138‑297 (MQ=255)`  
                                                                                                                                                                       `agagacagacacagtcacgcgctctggcgattcgctccaacgaactggctaacagtgcatccactaaagttaccgaggcggccagtggggcaaaccaagctcaacagctttccaaattctttgagtaatcaaaCAGGAGAAATCAATATGATCGTTTCTGACATCGAAGCTAACGCCCTCTTAGAGAGCGTCACTAAGTTCCACTGCGGGGTTATCTACGACTACTCCACCGCTGAGTACGTAAGCTACCGTCCGAGTGACTTCGGTGCGTATCTGGATGCGCTGGAAGCCGAGGTTGCAc                                                                                                                                       <  1:143123‑M2/168‑1 (MQ=255)`  
                                                                                                                                                                        `agaggccacacagtcacgcgctctggcgattcgctccaacgtactagctgacagtgcatccactaaagttaccgaggctgcccgtgtggcaaaccaagctcaactgctttccaaattctttgagtaatcaaaCAGGAGAAATCAATATGATCGTTTCTGACATCGAAGCTAACGCCCTCTTAGAGAGCGTCACTAAGTTCCACTGCGGGGTTATCTACGACTACTCCACCGCTGAGTACGTAAGCTACCGTCCGAGTGACTTCGGTGCGTATCTGGATGCGCTGGAAGCCGAGGTTGCACg                                                                                                                                      <  1:448004‑M2/169‑1 (MQ=255)`  
                                                                                                                                                                                      `tcacgcgctctggcgattcgctccaacgaactggctgacagtgcatccactaaagttaccgaggctgcccgtgtggcaaaccaagctcaacagctttccaaattctttgagtaatcatTAGGAGAAATCAATATGATCGTTTCTGACATCGAAGCTAACGCCCTCTTAGAGAGCGTCACTAAGTTCCACTGCGGGGTTATCTACGACTACTCCACCGCTGAGTACGTAAGCTACCGTCCGAGTGACTTCGGTGCGTATCTGGATGCGCTGGAAGCCGAGGTTGCACGAGGCGGTCTTATtg                                                                                                                        <  1:74625‑M2/183‑1 (MQ=255)`  
                                                                                                                                                                                      `tcacgcgctctggcgattcgctccaacgaactggctgacagtgcatccactaaagttaccgaggctgcccgtgtggcaaaccaagctcaacagctttccaaattctttgagtaatcaaTAGGAGAAATCAATATGATCGTTTCTGACATCGAAGCTAACGCCCTCTTAGAGAGCGTCACTAAGTTCCACTGCGGGGTTATCTACGACTACTCCACCGCTGAGTACGTAAGCTACCGTCCGAGTGACTTCGGTGCGTATCTGGATGCGCTGGAAGCCGAGGTTGCACGAGGCGGTCTTATtg                                                                                                                        <  1:525132‑M2/183‑1 (MQ=255)`  
                                                                                                                                                                                      `tcacgcgctctggcgattcgctccaacgaactggctgacagtgcatccactaaagttaccgaggctgcccgtgtggcaaaccaagctcaacagctttccaaattctttgagtaatcaaTAGGAGAAATCAATATGATCGTTTCTGACATCGAAGCTAACGCCCTCTTAGAGAGCGTCACTAAGTTCCACTGCGGGGTTATCTACGACTACTCCACCGCTGAGTACGTAAGCTACCGTCCGAGTGACTTCGGTGCGTATCTGGATGCGCTGGAAGCCGAGGTTGCACGAGGCGGTCTTATtg                                                                                                                        <  1:615436‑M2/183‑1 (MQ=255)`  
                                                                                                                                                                                      `tcacgcgcgctggcgattcgctcaaacgaactggctgacagtgcatccactaaagttaccgaggctgcccgtgtggaaaaccaagctcaacagctttccaaattctttgagtaatcaaTAGGAGAAATCAATATGATCGTTTCTGACATCGAAGCTAACGCCCTCTTAGAGAGCGTCACTAAGTTCCACTGCGGGGTTATCTACGACTACTCCACCGCTGAGTACGTAAGCTACCGTCCGAGTGACTTCGGTGCGTATCTGGATGCGCTGGAAGCCGAGGTTGCACGAGGCGGTCTTATtg                                                                                                                        <  1:490778‑M2/183‑1 (MQ=255)`  
                                                                                                                                                                                      `gcacgcgctctggcgattcgctccaacgaactggctgacagtgcatccactaaagttaccgaggctgcccgtgtggcaaaccaagctcaacagctttccaaattctttgagtaatcaaTAGGAGAAATCAATATGATCGTTTCTGACATCGAAGCTAACGCCCTCTTAGAGAGCGTCACTAAGTTCCACTGCGGGGTTATCTACGACTACTCCACCGCTGAGTACGTAAGCTACCGTCCGAGTGACTTCGGTGCGTATCTGGATGCGCTGGAAGCCGAGGTTGCACGAGGCGGTCTTATtg                                                                                                                        <  1:543216‑M2/183‑1 (MQ=255)`  
                                                                                                                                                                                      `cacgcgctctggcgattcgctccaacgaactggctgacagtgcatccactaaagttaccgaggctgcccgtgtggcaaaccaagctcaacagctttccaaattctttgagtaatcaaaCAGGAGAAATCAATATGATCGTTTCTGACATCGAAGCTAACGCCCTCTTAGAGAGCGTCACTAAGTTCCACTGCGGGGTTATCTACGACTACTCCTCCGCTGAGTACGTAAGCTACCGTCCGAGTGACTTCGGTGCGTATCTGGATGCGCTGGAAGCCGAGGTTGCACGAGGCGGTCTTATtg                                                                                                                        <  1:178427‑M2/183‑1 (MQ=255)`  
                                                                                                                                                                                      `cacgccctcgtgcgattcgctcaaacgaactggctgacagtgcatccactaaagttaccgaggctgcccgtgtggcaaaccaagctcaacagctttccaaattctttgagtaatcaaaCAGGAGAAATCAATATGATCGTTTCTGACATCGAAGCTAACGCCCTCTTAGAGAGCGTCACTAAGTTCCACTGCGGGGTTATCTACGACTACTCCACCGCTGAGTACGTAAGCTACCGTCCGAGTGACTTCGGTGCGTATCTGGATGCGCTGGAAGCCGAGGTTGCACGAGGCGGTCTTATtg                                                                                                                        <  1:376505‑M2/183‑1 (MQ=255)`  
                                                                                                                                                                                                 `ggcgattcgctccaacgaactggctgacagtgcatccactaaagttaccgaggctgcccgtgtggcaaaccaagctcaacagctttccaaattctttgagtaatcaaTAGGAGAAATCAATATGATCGTTTCTGACATCGAAGCTAACGCCCTCTTAGAGAGCGTCACTAATTTCCACTGCGGGGTTATCTACGACTACTCCACCGCTGAGTACGTAAGCTACCGTCCGAGTTACTTCGGTGCGTATCTGGATGCGCTGGAAGCCGAGGTTGCACGAggcggtcttattgctgtcctttat                                                                                                             >  1:2679‑M2/108‑290 (MQ=255)`  
                                                                                                                                                                                                    `attcgctccaacgaactggctgacagtgcatccactaaagttaccgaggctgcccgtgtggcaaaccaagctcaacagctttccaaattctttgagtaatcaaaCAGGAGAAATCAATATGATCGTTTCTGACATCGAAGCTAACGCCCTCTTAGAGAGCGTCACTAAGTTCCACTGCGGGGTTATCTACGACTACTCCACCGCTGAGTACGTAAGCTACCGTCCGAGTGACTTCGGTGCGTATCTGGATGCGCTGGAAGCCGAGGTTGCACGAGTCGGTCTTATTGTGTTCCACAACGGt                                                                                                          >  1:347194‑M2/105‑301 (MQ=255)`  
                                                                                                                                                                                                          `ctccaacgaactggcggaaagtgaatccaataaagttaccgaggcggcccgtgtggcaaaccaagctcaacagctttacaaattctttgagtaatcaaTAGGAGAAATCAATATGATCGTTTCTGACATCGAAGCTAACGCCCTCTTAGAGAGCGTCACTAAGTTCCGCTGCGGGGTTATCTACGACTACTCCACCGCTGAGTACGTAAGCTACCGTCCGAGTGACTTCGGTGCGTATCTGGATGCGCTGGAAGCCGAGGTTGCACGAGGCGGTCTTATTGTGTTCCACAACGGTCACAAg                                                                                                    <  1:458150‑M2/203‑1 (MQ=255)`  
                                                                                                                                                                                                          `ctccaacgaaagggttgacagtgcatcaactaaagttaccgaggctgaccgtgtggcaaaccaagatcaacagctttccaaattctgtgagtaatcaaTAGGAGAAATCAAGATGTTCGTTTCTGACATCGAAGCTAACGCCCTCTTAGAGAGCGTCACTAAGTTCCACTGCGGGGGTATCTAAGACTAATCCACCGCTGAGTACGTAAGCTACCGTCCGAGTGACTTAGGTGCGTATCTGGATGCGCTGGAAGCCGAGGTTGCACGAGGCGGTCTTATTGTGTTCCACAACGGTCACAAg                                                                                                    <  1:184577‑M2/203‑1 (MQ=255)`  
                                                                                                                                                                                                           `ccaacgaactggctgacagtgcatccactaaagttaccgaggctgccagtgtggcaaaccaagctcaacagcgttccaaattcattgagtaatcaaaCAGGATAAATCAATATGATCGTTTCTGACATCGAAGCTAACGCCCTCTTAGAGAGCGTAACTAAGTTCCACTGCGGGGTTATCTACGACTACTCCACCGCTGAGTACGTAAGCTACCGTCCGAGTGACTTCGGTGCGTATCTGGATGCGCTGGAAGCCGAGGTTGCACGAGGCGGTCTTATTGTGTTCCACAACGGTCACAAGt                                                                                                   <  1:190540‑M2/204‑1 (MQ=255)`  
                                                                                                                                                                                                               `gtgtataagagacaggtgcatccactaaagttaccgaggctgcccgtgtggcaaaccaagctcaacagctttccaaattctttgagtaatcaaTAGGAGAAATCAATATGATCGTTTCTGACATCGAAGCTAACGCCCTCTTAGAGAGCGTCACTAAGTTCCACTGCGGGGTTATCTACGACTACTCCACCGCTGAGTACGTAAGCTACCGTCCGAGTGACTTCGGTGCGTATCTGGATGCGCTGGAAGCCGAGGTTGCACGAGGCGGTCTTATTGTGTTCCACAACGGTCACAAGTATGa                                                                                               <  1:67974‑M2/208‑1 (MQ=255)`  
                                                                                                                                                                                                                   `actggctgacagtgcatccactaaagttaccgaggctgcccgtgtggcaaaccaagctcaacagctttccaaattctttgagtaatcaaTAGGAGAAATCAATATGATCGTTTCTGACATCGAAGCTAACGCCCTCTTAGAGAGCGTCACTAAGTTCCACTGCGGGGTTATCTACGACTACTCCACCGCTGAGTACGTAAGCTACTGTCCGAGTGACTTCGGTGCGTATCTGGATGCGCTGGAAGCCGAGGTTGCACGAGGCGGTCTTATTGTGTTCCACAACGGTCACAAGTATGACGtt                                                                                           <  1:292798‑M2/212‑1 (MQ=255)`  
                                                                                                                                                                                                                      `ggctgacagtgcatccactaaagttaccgaggctgcccgtgtggcaaaccaagctcaacagctttccaaattctttgagtaatcaaTAGGAGAAATCAATATGATCGTTTCTGACATCGAAGCTAACGCCCTCTTAGGGAGCGTCACTAAGTTCCACTGCGGGGTTATCTACGACTACTCCACCGCTGAGTACGTAAGCTACCGTCCGAGTGACTTCGGTGCGTATCTGGATGCGCTGGAAGCCGAGGTTGAACGGGGCGGTCTTATTGTGTTCCACAATGGTCACAAGTATGACGTGCCt                                                                                        >  1:280195‑M2/87‑301 (MQ=255)`  
                                                                                                                                                                                                                             `gtgcatccactaaagttaccgaggctgcccgtgtggcaaaccaagctcaacagctttccaaattctttgagtaatcaaaCAGGAGAAATCAATATGATCGTTTCTGACATCGAAGCTAACGCCCTCTTAGAGAGCGTCACTAAGTTCCACTGCGGGGTTATCTACGACTACTCCACCGCTGAGTACGTAAGCTACCGTCCGAGTGACTTCGGTGCGTATCTGGATGCGCTGGAAGCCGAGGATGCACGAGGCGGTCTTATTGTGTTCCACAACGGTCACAAGTATGACGTTCCTGCATTGa                                                                                 >  1:322479‑M2/80‑301 (MQ=255)`  
                                                                                                                                                                                                                                         `aaagttaccgaggctgcccgtgtggcaaaccaagctcaacagctttccaaattctttgagtaatcaaTAGGAGAAATCAATATGATCGTTTCTGACATCCAAGCTAACGCCCTCTTAGAGAGCGTCACTAAGTTCCACTGCGGGGTTATCTACGACTACTCCACCGCTGAGTACGTAAGCTACCGTCCGAGTGACTTCGGTGCGTATCTGGATGCGCTGGAAGCCGAGGTTGAACGAGGCGGTCTTATTTTGTTCAACAACGGTCCCAAGTCTGCCGTTCCTGCATTGACCACACTGGCaa                                                                     >  1:441187‑M2/68‑301 (MQ=255)`  
                                                                                                                                                                                                                                              `taccgaggctgcccgtgtggcagaccaatctcaacagctttccaaattctttgagtaatcaaTAGGAGAAATCAATATGATCGTTTCTGACATCTAAGCTAACGCCCTCTTAGAGAGCGTCACTAAGTTCCACTGCGGGGTTGTCTACGACTACTCCACCGCTGAGTACGTAAGCTACCGTCCGAGTGACTTCGGTGCGTATCTGGATGCGCTGGAAGCCGAGGTTGCACGAGGCGGTCTTATTGTGTTCCACAACGGTCACAAGTATGACGTTCCTGCATTGACCAAACTGGCAAAGTTg                                                                >  1:196763‑M2/63‑301 (MQ=255)`  
                                                                                                                                                                                                                                                       `tgcccgtgtggcaaaccaagctcaacagctttccaaattctttgagtaatcaaTAGGAGAAATCAATATGATCGTTTCTGACATCGAAGCTAACGACCTCTTAGAGAGCGTCACTAAGTTCCACTGCGGGGTTATCTACGACTACTCCACCGCTGAGTACGTAAGCTACCGTCCGAGTGACTTCGGTGCGTATCTGGATGCGCTGGAAGCCGAGGTTGCACGAGGCGGTCTTATTGTGTTCCACAACGGTCACAAGTATGACGTTCCTGCATTGACCAAACTGGCAAAGTTGCAATTGAAc                                                       <  1:50960‑M2/248‑1 (MQ=255)`  
                                                                                                                                                                                                                                                            `gtgtggcaaaccacgctcaacagctttccaaattctttgagtaatcaaTAGGAGAAATCAATATGATCGTTTCTGACATCGAAGCTAACGCCCTCTTAGAGAGCGTCACTAAGTTCCACTGCGGGGTTATCTACGACTACTCCACCGCTGAGTACGTAAGCTACCGTCCGAGTGACTTCGGTGCGTATCTGGATGCGCTGGAAGCCGAGGTTGCACGAGGCGGTCTTATTGTGTTCCACAATGGTCACAATTATGACGTTCCTGCATTGACCAAACTGGCAAAGTTGCAATTGAACCgaga                                                  >  1:435678‑M2/49‑301 (MQ=255)`  
                                                                                                                                                                                                                                                            `gtgtggcaaaccaagctcaacagctttccaaattctttgagtaatcaaTAGGAGAAATCAATATGATCGTTTCTGACATCGAAGCTAACGCCCTCTTAGAGAGCGTCACTAAGTTCCACTGCGGGGTTATCTACGACTACTCCACCGCTGAGTACGTAAGCTACCGTCCGAGTGACTTCGGTGCGTATCTGGATGCGCTGGAAGCCGAGGTTGCACGAGGCGGTCTTATTGTGTTCCACAACGGTCACAAGTATGACGTTCCTGCATTGACCAAACTGGCAAAGTTGCAATTGAACCgaga                                                  <  1:225562‑M2/253‑1 (MQ=255)`  
                                                                                                                                                                                                                                                              `tggcaaaccaagctcaacagctttccaaattctttgagtaatcaaaCAGGAGAAATCAATATGATCGTTTCTGACATCGAAGCTAACGCCCTCTTAGAGAGCGTCACTAAGTTCCACTGCGGGGTTATCTACGACTACTCCACCGCTGAGTACGTAAGCTACCGTCCGAGTGACTTCGGTGCGTATCTGGATGCGCTGGAAGCCGAGGTTGCACGAGGCGGTCTTATTGTGTTCCACAACGGTCACAAGTATGACGTTCCTGCATTGACCAAACTGGCAAAGTTGCAATTGAACCGAGAGt                                                >  1:610151‑M2/47‑301 (MQ=255)`  
                                                                                                                                                                                                                                                                `ggcaaaccaagctcaacagctttccaaattctttgagtaatcaaTAGGAGAAATCAATATGATCGTTTCTGACATCGAAGCTAACTCCCTCTTATAGAGCGTCACTAAGTTCCACTGCGGGGTTATCTACGACTACTCCACCGCTGAGTACGTAAGCTACCGTCCGAGTGACTTCGGTGCGTATCTGTATGCGCTGGAATCCGAGGTTGCACGAGGCGGCATTAATTTGTTCCACAACGGTCCCAAGTATGACGTTCCTGAATTGACCAAACTGGCAAAGTTGCAATTGacccgagatctc                                              >  1:327612‑M2/45‑297 (MQ=255)`  
                                                                                                                                                                                                                                                                   `aaccaagctcaacagctttccaaatgctttgagtaatcaaaCAGGAGAAATCAATATGATCGTTTCTGACATCGAAGCTAACGCCCTCTTAGAGAGCGTCACTAAGTTCAACTGCGGGGTTATCTACGACTACTCCACCGCTGAGTACGTAAGCTACCGTCCGAGTGACTTCGGTGCGTATCTGGATGCGCTGGAAGCCGAGGTTGCACGAGGCGGTCTTATTGTGTTCCACAACGGTCACAAGTATGACGTTCCTGCGTTGACCAAACTGGCAAAGTTGCAATTGAACCGAGAGTTCCAc                                           <  1:255215‑M2/260‑1 (MQ=255)`  
                                                                                                                                                                                                                                                                   `aaaccaagctcaacagctttccaaattctttgagtaatcaaTAGGAGAAATCAATATGATCGTTTCTGACATCGAAGCTAACGCCCTCTTAGAGAGCGTCACTAAGTTCCACTGCGGGGTTATCTACGACTACTCCACCGCTGAGTACGTAAGCTACCGTCCGAGTGACTTCGGTGCGTATCTGGATGCGCTGGAAGCCGAGGTTGCACGAGGCGGTCTTATTGTGTTCCACAACGGTCACAAGTATGACGTTCCTGCATTGACCAAACTGGCAAAGTTGCAATTGAACCGAGAGTTCCAc                                           <  1:472342‑M2/260‑1 (MQ=255)`  
                                                                                                                                                                                                                                                                   `aaaccaagctcaacagcgttccaaattctttgagtaatcaaTAGGAGAAATCAATATGATCGTTTCTGACATCGAAGCTAACGCCCTCTTAGAGAGCGTCACTAAGTTCCACTGCGGGGTTATCTACGACTACTCCACCGCTGAGTACGTAAGCTACAGTCCGAGTGACTTCGGTGCGTATCTGGATGCGCTGGAAGCCGAGGTTGCACGAGGCGGTCTTATTGTGTTCCACAACGGTCACAAGTATGACGTTCCTGCATTGACCAAACTGGCAAAGTTGCAATTGAACCGAGAGTTCCAc                                           <  1:412032‑M2/260‑1 (MQ=255)`  
                                                                                                                                                                                                                                                                                       `caaatgctttgagtaatcaaaCAGGATAAATCAATATGATCGTTTCTGACATCGAAGCTAACGCCCTCTTAGAGAGCGTCACTAAGTTCCACTGCAGGGTTATCTACGACTACTCCACCGCTGAGTACGTAAGCTACTGTCCGAGTGACTTCGGTGCGTATCTGGATGCGCTGGAAGCCGAGGTTGCACGAGGCGGTCTTATTGTGTTCCACAACGGTCACAAGTATGACGTTCCTGCATTGACCAAACTGGCAAAGTTGCAATTGAACCGAGAGTTCCACCTTCCTCGTGAGAACTGTAt                       <  1:93083‑M2/280‑1 (MQ=255)`  
                                                                                                                                                                                                                                                                                       `caaatgctttgagtaatcaaaCAGGAGAAATCAATATGATCGTTTCTGACATCGAAGCTAACGCCCTCTTAGAGAGCGTCACTAAGTTCCACTGCGGGGTTATCTACGACTACTCCACCGCTGAGTACGTAAGCTACCGTCCGAGTGACTTCGGTGCGTATCTGGATGCGCTGGAAGCCGAGGTTGCACGAGGCGGTCTTATTGTGTTCCACAACGGTCACAAGTATGACGTTCCTGCATTGACCAAACTGGCAAAGTTGCAATTGAACCGAGAGTTCCACCTTCCTCGTGAGAACTGTAt                       <  1:244077‑M2/280‑1 (MQ=255)`  
                                                                                                                                                                                                                                                                                       `aaaattctttgaggaatcaaaCAGGAGAAATCAATATGATCGTTTCTGACATCGAAGCTAACGCCCGCTGAGAGAGCGTCACTAAGTTCCACTGCGGGGTTATCTACGACTACTCCACCGCTGAGTACGTAAGCTACCGTCCGAGTGACTTCGGTGCGTATCTGGACGCGCTGGAAGCCGAGGTTGCACGAGGCGGTCTTATCGTGTTCCACAACGGTCACAAGTATGACGTTCCTGCATTGACCAAACTGGCAAAGTTGCAATTGAACCGAGAGTTCCACCTTCCTCGTGGGAACTGTAt                       <  1:596432‑M2/280‑1 (MQ=255)`  
                                                                                                                                                                                                                                                                                           `ttctttgagtaatcaaaCAGGAGAAATCAATATGATCGTTTCTGACATCGAAGCTAACGCCCTCTTAGAGAGCGTCACTAAGTTCCACTGCGGGGTTATCTACGACTACTCCACCGCTGAGTACGTAAGCTACCGTCCGAGTGACTTCGGTGCGTATCTGGATGCGCTGGAAGCCGAGGTTGCACGAGGCGGTCTTATTGTGTTCCACAACGGTCACAAGTATGACGTTCCTGCATTGACCAACCTGGCAAAGTTGCAATTGAACCGAGAGTTCCACCTTCCTCGTGAGAACTGTATTGac                   >  1:551739‑M2/18‑301 (MQ=255)`  
                                                                                                                                                                                                                                                                                            `ttctttgagtaatcaaTAGGAGAAATCAATATGATCGTTTCTGACATCGAAGCTAACGACCTCTTAGAGAGCGTCACTAAGTTCCACTGCGGGGTTATCTACGACTACTCCACCGCTGAGTACGTAAGCTACCGTCCGAGTGACTTCGGTGCGTATCTGGATGCGCTGGAAGCCGAGGTTGCACGAGGCGGTCTTATTGTGTTCCACAACGGTCACAAGTATGACGTTCCTGCATTGACCAAACTGGCAAAGTTGCAATTGAACCGAGAGTTCCACCTTCCTCGTGAGAACTGTATTGaca                  <  1:642754‑M2/285‑1 (MQ=255)`  
                                                                                                                                                                                                                                                                                             `ctttgagtaatcaaaCAGGAGAAACCATTATGATCGTTTCTGACATCGAAGCTAACGCCCTCTTATAGAGCGTCACTAAGTTCCACTGCGGGGTTATCTACGACTACTCCACCGCTGAGTACGTAAGCTACCGTCCGAGTGACTTCGGTGCGTATCTGGATGCGCTGGAAGCCGAGGTTGCACGAGGCGGTCTTATTGTGTTCCACAACGGTCACAAGTATGACGTTCCTGCATTGACCAAACTGGCAAAGTTGCAATTGAACCGAGAGTTCCACCTTCCTCGTGAGAACTGTATTGacac                 <  1:495150‑M2/286‑1 (MQ=255)`  
                                                                                                                                                                                                                                                                                              `tttgagtaatcaaaCAGGAGAAACCATTATGATCGTTTCTGACATCGAAGCTAACGCCCTCTTAGAGAGCGTCACTAAGTTCCACGGCGGGGTTATCTACGACTACTCCACCGCTGAGTACGTAAGCTACCGTCCGAGTGACTTCGGTGCGTATCTGGATGCGCTGGAAGCCGAGGTTGCACGAGGCGGTCTTATTGTGTTCCACAACGGTCACAAGTATGACGTTCCTGCATTGACCAAACTGGCAAAGTTGCAATTGAACCTAGAGTTCCACCTTCCTCGTGAGAACTGTATTGACAcc                <  1:105401‑M2/287‑1 (MQ=255)`  
                                                                                                                                                                                                                                                                                                  `agacagtcaaTAGGAGAAATCAATATGATCGTTTCTGACATCGAAGCTAACGCCCTCTTAGAGAGCGTCACTAAGTTCCACTGCGGGGTTATCTACGACTACTCCACCGCTGAGTACGTAAGCTACCGTCCGAGTGACTTCGGTGCGTATCTGGATGCGCTGGAAGCCGAGGTTGCACGAGGCGGTCTTATTGTGTTCCACAACGGTCACAAGTATGACGTTCCTGCATTGACCAAACTGGCAAAGTTGCAATTGAACCGAGAGTTCCACCTTCCTCGTGAGAACTGTATTGACACCCTtg            <  1:23210‑M2/291‑1 (MQ=255)`  
                                                                                                                                                                                                                                                                                                     `taatcaaTAGGAGAAATAAATATGATCGTTTCAGACATCGAAGCTAACGCCCTCTTAGAGAGCGTCACTAAGTTCCACTGAGGTGTTATCTACGACTACTCCACCGCTGAGTACGTAAGCTACCGTCCGAGTGACTTCGGTGCGTATCTGGATGCGCTGGAAGCCGAGTTTGCACGAGGCGGTCTTATTGTGTTCCACAACGGTCACAAGTATGACGTTCCTGAATTGACCAAACTGGCAAAGTTGCAATTGAACCGAGAGTTCCACCTTCCTCGTGAGAACTGTATTGACACCCTTgtgt         <  1:305067‑M2/294‑1 (MQ=255)`  
                                                                                                                                                                                                                                                                                                       `atcaaTAGGAGAAATCAATATGATCGTTTCTGACATCGAAGCTAACGCCCTCTTAGAGAGCGTCACTAAGTTCCACTGCGGGGTTATCTACGACTACTCCACCGCTGAGTACGTAAGCTACCGTCCGAGTGACTTCGGTGCGTATCTGGATGCGCTGGAAGCCGAGGTTGCACGAGGCGGTCTTATTGTGTTCCACAACGGTCACAAGTATGACGTTCCTGCATTGACCAAACTGGCAAAGTTGCAATTGAACCGAGAgttccaccttcctcgtgagaacctgtctcttatacccatctcc       >  1:80189‑M2/6‑280 (MQ=255)`  
                                                                                                                                                                                                                                                                                                       `atcaaTAGGAGAAATCAATATGATCGTTTCTGACATCGAAGCTAACGCCCTCTTAGAGAGCGTCACTAAGTTCCACTGCGGGGTTATCTACGACTACTCCACCGCTGAGTACGTAAGCTACCGTCCGAGTGACTTCGGTGCGTATCTGGATGCGCTGGAAGCCGAGGTTGCACGAGGCGGTCTTATTGTGTTCCACAACGGTCACAAGTATGACGTTCCTGCATTGACCAAACTGGCAAAGTTGCAATTGAACCGAGAGTTCCACCTTCCTCGTGAGAACTGTATTGACACCCTTgtgttt       >  1:465935‑M2/6‑300 (MQ=255)`  

                                                                                                                                                                                                                                                                                                            `|`                                                                                                                                                                                                                                                                                                              
`TAGGCTGGACTTGGACAAGACAGACTGGCTGGAAGAAACCAGAAGTTACCAAGCTGAGTCATAAGGTGTTCGATAAGGACACTATGACCCACATCAAGGCTGGTGATTGGGTTAAGGTTGACATGGGAGTTGTTGGTGGATACGGCTACGTCCGCTCAGTTAGTGGCAAATATGCACAAGTGTCATACATCACAGGTGTTACTCCACGCGGTGCAATCGTTGCCGATAAGACCAACATGATTCACACAGGTTTCTTGACAGTTGTTTCATATGAAGAGATTGTTAAGTCACGATAATCAATAGGAGAAATCAATATGATCGTTTCTGACATCGAAGCTAACGCCCTCTTAGAGAGCGTCACTAAGTTCCACTGCGGGGTTATCTACGACTACTCCACCGCTGAGTACGTAAGCTACCGTCCGAGTGACTTCGGTGCGTATCTGGATGCGCTGGAAGCCGAGGTTGCACGAGGCGGTCTTATTGTGTTCCACAACGGTCACAAGTATGACGTTCCTGCATTGACCAAACTGGCAAAGTTGCAATTGAACCGAGAGTTCCACCTTCCTCGTGAGAACTGTATTGACACCCTTGTGTTGTCACG  >  NC_001604.1/14039‑14639`

  

Alignment Legend

Aligned base mismatch/match (shaded by quality score): `ATCG/ATCG` `< 3 ≤` `ATCG/ATCG` `< 10 ≤` `ATCG/ATCG` `< 21 ≤` `ATCG/ATCG` `< 34 ≤` `ATCG/ATCG` `< 38 ≤` `ATCG/ATCG`

Unaligned base: `atcg`    Masked matching base: `atcg`    Alignment gap: `‑`    Deleted base: `‑`

Reads not counted as support for junction

read\_name Not counted due to insufficient overlap past the breakpoint.

read\_name Not counted due to not crossing MOB target site duplication.
